# A Systematic Review and Meta-Analysis of the Effect of Lifestyle Modification on Metabolic Control in Overweight Children

**DOI:** 10.1155/2017/5681909

**Published:** 2017-09-27

**Authors:** Angela Shin-Yu Lien, Jia-Ling Tsai, Jian-Tao Lee, Mei-Yao Wu, Yi-Der Jiang, Hung-Rong Yen

**Affiliations:** ^1^School of Nursing, College of Medicine, Chang Gung University, Taoyuan, Taiwan; ^2^Division of Endocrinology and Metabolism, Department of Internal Medicine, Chang Gung Memorial Hospital, Linkou Branch, Taoyuan, Taiwan; ^3^School of Nursing, College of Medicine, National Taiwan University, Taipei, Taiwan; ^4^Research Center for Traditional Chinese Medicine, Department of Medical Research, China Medical University Hospital, Taichung, Taiwan; ^5^Department of Internal Medicine, National Taiwan University Hospital, Taipei, Taiwan; ^6^Department of Chinese Medicine, China Medical University Hospital, Taichung, Taiwan; ^7^Research Center for Chinese Herbal Medicine, China Medical University, Taichung, Taiwan; ^8^School of Chinese Medicine, China Medical University, Taichung, Taiwan; ^9^Department of Biotechnology, Asia University, Taichung, Taiwan

## Abstract

Childhood obesity is associated with type 2 diabetes mellitus. We aimed to determine the effects of lifestyle modification programs on fasting plasma glucose (FPG) levels in overweight children. We queried six relevant electronic databases and manually searched for studies published before December 2016. Overweight/obese children who underwent a lifestyle modification for more than 6 months were included. A total of 3923 children from eight randomized controlled trials (RCTs) were included. Compared with the control group, the lifestyle modification group had significantly lower FPG levels by 1.3 mg/dL. The mean differences were significantly decreased for both secondary outcomes; BMI *z*-score decreased by 0.16 units and insulin levels decreased by 2.4 mU/L. The metaregression showed that the follow-up duration was associated with FPG levels and BMI and insulin levels and half year is a suitable follow-up duration for this population. This study showed that lifestyle modification programs may be effective in reducing the FPG levels of overweight/obese children. Further high-quality RCTs with longer follow-up periods are needed to evaluate the long-term effect of this complementary approach for diabetes mellitus prevention on overweight/obese children.

## 1. Introduction

Childhood obesity is one of the most important health challenges that pose long-term impacts on medical, psychosocial, and financial consequences [1, 2]. In 2010, the estimated number of overweight children under 5 years old was more than 42 million worldwide. Nearly 35 million of these children lived in developing countries, mostly in urban settings [3]. The prevalence of overweight children between 5 and 12 years old in the United States, Italy, Greece, and Spain is greater than 30% [4]. An increased prevalence of overweight children has also been identified in Taiwan. The prevalence of overweight Taiwanese children aged 6 to 12 years was 29.8% for boys and 25.1% for girls [5].

Type 2 diabetes was known as an “adult-onset” disease. However, the increasing prevalence of type 2 diabetes in children parallels the increasing incidence of obesity worldwide. Overweight and obese youth are also associated with cardiovascular disease and type 2 diabetes due to the accumulation of body fat, which is highly correlated with fasting insulin release [6]. In 2000, nearly 45% of new pediatric cases with type 2 diabetes were overweight, sharply increasing from 4% in the 1990s [2]. According to the HEALTHY study that involved 6,358 sixth-grade pupils, 49.3% of children had a body mass index (BMI) in the 85th percentile or higher, and 16.0% demonstrated fasting plasma glucose (FPG) levels greater than 100 mg/dL even though they did not have signs and symptoms of diabetes [7]. Type 2 diabetes is highly associated with obesity and plasma glucose level. Race (particularly minority populations), obesity, and family history were prominent risk factors related to adolescent type 2 diabetes [8]. An increased waist circumference was a strong risk factor for type 2 diabetes as well [6]. In a nationwide urine-screening program for nearly 3 million school children in Taiwan, the odds of type 2 diabetes mellitus were 18.8 times higher for those with a BMI over the 95th percentile relative to those with a BMI below the 50th percentile [9]. Mortality, atherosclerotic cardiovascular disease, and complication rates were higher for patients with young type 2 than for those with type 1 diabetes [10].

Strategies to control body weight and to address the related complications of obesity are important issues for pediatric healthcare. In an ecological context, unhealthy behavior, lifestyle, and environmental issues all contribute to childhood obesity. Therefore, lifestyle modification has been applied as complementary therapy to prevent children from obesity, diabetes, and cardiovascular diseases. Increased sedentary behavior, an energy imbalance, an excessive caloric intake, and poor dietary components are major elements of lifestyle that contribute to obesity [11]. Pharmaceutical and surgical therapies may be helpful to quickly reduce extreme body weight, but lifestyle interventions that encourage an increase in physical activity and the consumption of healthy foods are essential to maintain a long-term behavior change for better type 2 diabetes management [12]. In the United Kingdom Prospective Diabetes Study (UKPDS), diabetic patients improved glycemic control and decreased other cardiovascular risk factors through standard lifestyle regulation or adjunctive medical or surgical interventions for severe hyperglycemia [13]. Overweight/obesity not only contributes to poor health but also affects the psychological and social well-being of children. Furthermore, it affects the economy. Over the last 20 years, annual obesity-related hospital costs have increased 3-fold, reaching $127 million per year in the United States [10]. However, despite its impact on health and psychological and social well-being and economic impact, obesity can be prevented through early lifestyle modifications in childhood [14].

In the Western countries, “complementary and alternative” usually refer to the context in which a therapy is used. So a therapy can be complementary if it is used alongside conventional treatments or alternative if it is used in place of a conventional treatment. Lifestyle modifications, including a healthy diet, weight control, increased physical activity, and reduced sedentary behavior, have been recommended as a complementary therapy for the management of obese and hyperglycemic subjects to prevent or delay the onset of type 2 diabetes [15] and are consistent with evidence gathered from adult populations [16–21]. The American Diabetes Association recently revised its strategic plan for lifestyle modification; it called for increasing focus on the translation of prevention research into strategies for primary care and community treatment to reduce the risk of developing type 2 diabetes [22, 23].

Many clinical trials have been conducted in schools, hospitals, families, and communities but have led to mixed results [24, 25]. Some trials effectively improved knowledge or skills, but most failed to prevent obesity or decrease blood glucose, insulin, and other risk factors in children. A randomized, controlled pilot study indicated that Exenatide® can significantly reduce body mass index for extreme obesity, but it cannot reduce FPG [23]. Moderate to vigorous physical activity can lower waist circumference, blood pressure, insulin level, and triglycerides in children and adolescents [26]. However, the effects of lifestyle modifications, the most important nonpharmaceutical approach, on glucose control or diabetes prevention have not been well established. In addition, studies on youth have not utilized impaired glucose tolerance (IGT) as a marker of progression toward type 2 diabetes, and the long-term effectiveness of obesity treatment and prevention programs provided for children need to be clarified. To fill the gap, we conducted a systematic review that searched for lifestyle modifications as the intervention in clinical trials. Then, we applied meta-analytical methods to determine the effect of lifestyle modifications on FPG as the primary outcome and BMI or BMI *z*-score, fasting insulin level, and HbA_1C_ as the secondary indicators among overweight/obese children to make a conclusion.

## 2. Material and Methods

### 2.1. Definition of Overweight and Obesity in Children

The World Health Organization (WHO) has suggested the use of BMI as an indicator of extreme body weight. BMI is a simple index of the weight-to-height ratio (kg/m^2^) that been used commonly to classify individuals as underweight, overweight, or obese. This study adopted the definition of the International Obesity Task Force (IOTF) and the references of the WHO to classify individuals as underweight, overweight, or obese [28, 29]. An overweight child has a BMI that falls between the 85th and 95th percentile after adjustment for gender and age; this is approximately equivalent to the BMI range from 25 to 29.99 kg/m^2^ in adults (the normal range for adults is 18.5 to 24.99 kg/m^2^; for Asian adults, the cut-off point is 23). Likewise, an obese youth has a BMI that lies above the 95th percentile, equivalent to at least 30 kg/m^2^ in adults (for Asian adults, the cut-off point is 27, and it considers age and gender) [30].

### 2.2. Study Strategy and Eligibility Criteria

This review integrated the results from relevant studies by following the systematic review and meta-analysis guidelines outlined in the Preferred Reporting Items for Systematic Review and Meta-Analysis (PRISMA) statement [31]. The authors conducted a comprehensive literature search of the PubMed, Cochrane, EMBASE, Cinahl, ProQuest dissertation and thesis, and PsychINFO databases and manually searched for studies published before December 2016. There were no limitations based on language, and the search focused on the influence of lifestyle interventions (more than 6 months) on FPG levels in overweight/obese children. The search strategy included the following keywords and MeSH terms: “randomized control trial” OR “clinical control trial” AND “childhood overweight and obesity” AND “lifestyle,” AND “diabetes” AND “exercise” OR “physical activity” OR “fitness.” Studies that evaluated subjects with certain diseases or conditions (e.g., neurosurgical, cardiovascular, diabetes mellitus, eating disorders, malnutrition, and psychosocial or physical disabilities) were excluded, along with those that involved the participation of subjects in interventions other than lifestyle modification (e.g., breastfeeding) or that provided pharmacological treatment for obesity.

Using this method, we identified 572 articles, including 5 relevant systematic reviews retrieved by manual search. After 332 duplicates studies were removed, the abstracts of all of the identified articles were reviewed independently by 2 authors. Overweight children under the age of 18 were included in the review. The intervention was limited to lifestyle modification programs that included diet control, healthy nutrition, exercise, fitness, and/or physical activities. The primary outcome was FPG levels, and the secondary outcome indicators were BMI or BMI *z*-scores, fasting insulin level, and HbA_1C_. Furthermore, an article was excluded if the full text was not available or if it was not an original study.

**Figure 1:**
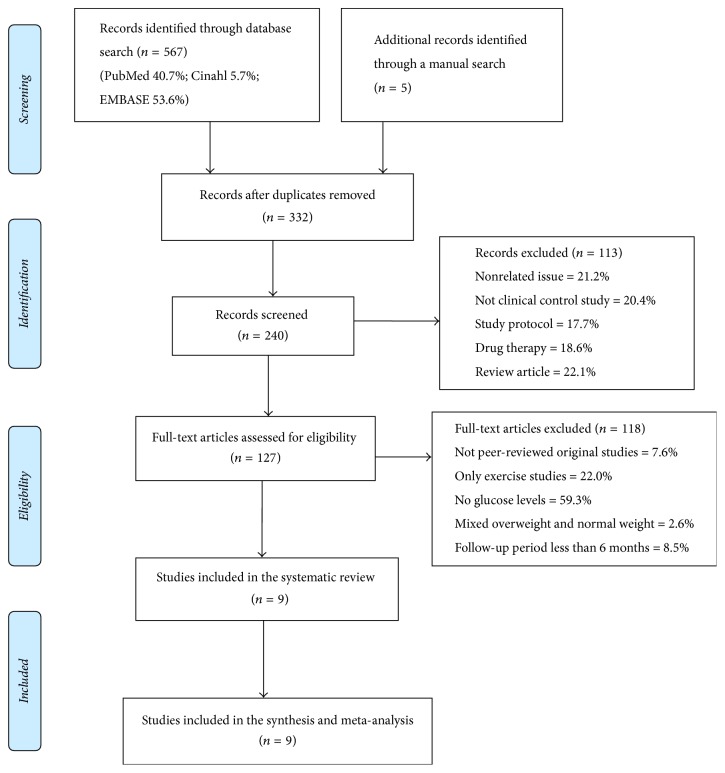
The study selection and screening diagram. From [27].

After these criteria were applied, 127 articles with full text remained, of which we excluded 70 studies that did not report FPG, 26 studies that included lifestyle programs which only provided an exercise intervention, 9 studies that were not peer-reviewed original studies, 10 studies that had a follow-up period less than 6 months or that fulfilled other exclusion criteria, and 3 studies that mixed both normal weight and overweight children [32–34]. The remaining 9 studies were included in the qualitative synthesis and meta-analysis [35–43]. The full texts of the included articles were reviewed independently by 2 authors, and disagreements were resolved by consensus. A detailed screening diagram is shown in Figure 1.

### 2.3. Data Extraction

The 9 studies included in the meta-analysis were abstracted systematically for details and characteristics, including study location, design, subjects, intervention style (i.e., lifestyle modification program), and study period (Table 1). The primary outcome of FPG and the secondary outcomes of BMI or BMI *z*-score, insulin level, and HbA_1C_ were taken from the references of the included articles (Tables 2 and 3). During the systematic review, 2 reviewers extracted data independently using a standardized data extraction form. To resolve assessment differences, the reviewers deliberated until a consensus was reached.

### 2.4. Quality Assessment

We carefully reviewed the design, conduct, analysis, and reporting of each study and considered whether there was any risk of bias that would make the 2 groups noncomparable. We used a domain-based approach with Cochrane risk of bias tool [44] to assess the quality of each included study. It contains the following domains: (1) random sequence generation, (2) allocation concealment, (3) blinding of participants, (4) personnel blinding of outcome assessment, (5) incomplete outcome data, (6) selective outcome reporting, and (7) other sources of bias (see Figure S1 in Supplementary Material available online at https://doi.org/10.1155/2017/5681909). The judgment of whether there was high, low, or unknown bias was reached by consensus between 2 reviewers.

### 2.5. Analysis and Synthesis Methods

Qualitative synthesis and the random-effects model meta-analysis were performed using Comprehensive Meta-Analysis (CMA) software Version 2.2; it combined studies under the random-effects model and compared the mean changes in FPG levels between the intervention and control groups [45]. We calculated difference in means and 95% confidence intervals (CI) for estimate the effect between lifestyle intervention and control for overweight/obese children.

## 3. Results

### 3.1. Study Population Characteristics

In total, there were 3923 subjects, of whom 1943 subjects were in the intervention group and 1980 in the control group. Five studies were performed in the United States with subjects aged 9~14 years (one for Mexican-Americans and one for Latinos), two were in the Netherlands with subjects aged 8~17, and the remained 2 studies were one from West Germany [40] and one was from China [43]. Six studies recruited 41~81 children in a single center with a treatment lasting within 6~12 months, one study collects data from school cluster sampling [43], and only one multiple center study was performed as part of the HEALTHY study [37], which was a much larger study that recruited more than a thousand overweight kids in each group with a treatment lasting within 1~3 years (Table 1). But none of any included studies report the issue of gender difference.

### 3.2. Outcome Indicators

For fasting glucose levels, for the primary outcome, the results are summarized in Table 2. One study showed no change in either the intervention or control group. Five studies showed greater FPG reduction in the intervention group than in the control group; however, none of the differences were statistically significant. Three articles showed a decrease in FPG for the intervention group and an increase for the control group. Two articles reported a decrease in FPG for both groups, and three studies reported an increase in FPG for both groups. Regarding BMI *z*-scores, four studies showed a decrease for both the intervention and control groups, two articles showed a decrease for the intervention group and an increase for the control group, one article showed no change for the intervention group but a decrease for the control group, and the remaining study showed an increase for the intervention group and no change in the control group. Regarding insulin level changes, four articles reported an increase for both the intervention and control groups, two articles reported a decrease for both groups, one article reported a decrease for the intervention group but an increase for the control group, the last one report no data. Among the 9 studies, only three of them reported HbA_1C_ level (Table 3).

Regarding other biomarkers, two articles showed changes in the insulin resistance index (homeostasis model assessment, HOMA) for both groups. Seven studies reported changes in the lipid profile (triglyceride, high density lipoprotein, and low density lipoprotein), of which one showed an inverse effect on both groups and two found no effect on either group.

### 3.3. Meta-Analysis

**Figure 2:**
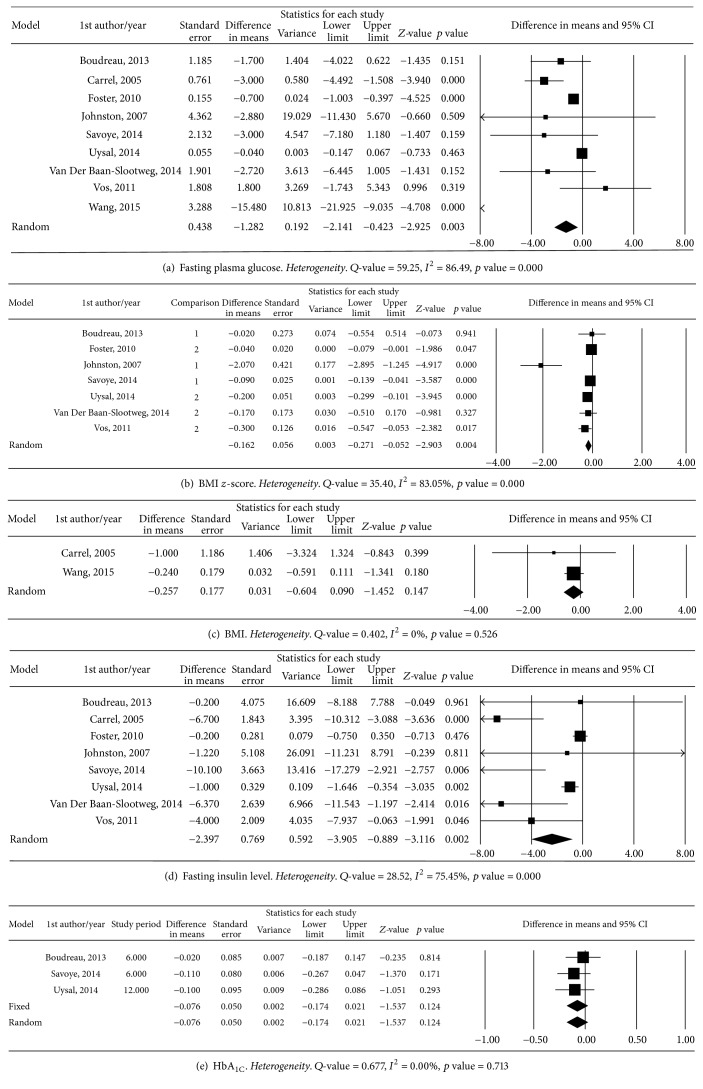
Meta-analysis of the effect size by difference in means.

We pooled all of the studies into a meta-analysis, and the forest plot of the random-effects model showed moderate heterogeneity, as demonstrated by the high *I*^2^ value (86.49%). The point estimate of the mean difference was −1.282 mg/dL (95% CI, −2.14 to −0.42, *Z* = −2.92, *p* = 0.003), which indicated that lifestyle modifications decreased the mean fasting glucose levels by 1.3 mg/dL. The sensitivity test indicated that the omission of any study would change the estimate between −0.66 (−11.43 to 5.60) mg/dL and −4.71 (−21.93 to −9.04) mg/dL. In addition, we analyzed the change in the secondary outcome variables of BMI *z*-score and insulin level among the seven studies. After the data were pooled for the meta-analysis, the effect sizes of the mean differences in BMI *z*-scores and insulin levels were 0.06 units (95% CI = −0.27 to −0.05, *Z* = −2.90, *p* = 0.004) and −2.4 mU/L (95% CI = −3.91 to −0.89, *Z* = −3.12, *p* = 0.002). The results indicated a significant decrease in the secondary outcome variables due to lifestyle interventions between the 2 groups from baseline to the end of the study. We also found that the change of HbA_1C_ level was −0.08%. However, there was no significant difference (95% CI = −0.17 to 0.21, *Z* = −1.54, *p* = 0.12) (Figure 2).

The results from both asymmetry funnel plot and Egger's linear regression test (*t* = 2.55, *p* = 0.004) showed significant publication bias of all eligible studies (Figure S2).

**Figure 3:**
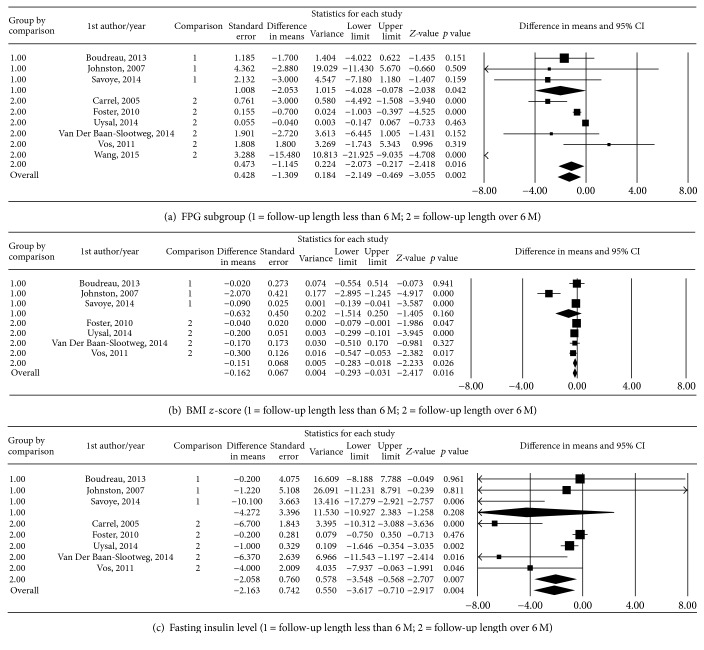
Meta-analysis forest plot of the mean changes according to follow-up length: group 1, indicating less than 6 months; group 2, indicating longer than 6 months.

The high *I*^2^ values for the FPG (86.49%, Egger's value, *t* = 2.55, *p* = 0.04), BMI *z*-score (83.05% Egger's value, *t* = 2.29, *p* = 0.07), and insulin level (75.45%, Egger's value, *t* = 2.69, *p* = 0.04) analyses indicated heterogeneity (Figure S2), and the meta-regression showed an association of these variables with follow-up length (Figure S3). A further subgroup analysis was performed dividing the studies into 2 groups according to follow-up periods: group 1 for 6 months and group 2 between 9 months and 3 years. The results are shown in Figure 3. Lifestyle interventions significantly reduced FPG by 1.31 mg/dL (95% CI, −2.15 to −0.47, *Z* = −3.06, *p* = 0.002), BMI *z*-scores by −0.16 (95% CI, −0.29 to −2.42, *Z* = −0.03, *p* = 0.016), and insulin levels by −2.16 mU/L (95% CI, −3.62 to −0.71, *Z* = −2.92, *p* = 0.004) in the subgroup analysis of the random-effects model (Figure 3).

## 4. Discussion

The present meta-analysis reported that patients who received lifestyle modification showed significantly reduced FPG levels by 1.3 mg/dL compared with patients in the control group. This effect indicated that lifestyle modification interventions could decrease FPG levels, BMI, and insulin levels in overweight/obese children. In adults with IGT or impaired fasting glucose (IFG), lifestyle modification has consistently shown positive results. Previous studies have reported that lifestyle modification programs significantly reduced BMI, body fat, waist circumference, blood sugar levels, glycated hemoglobin (HbA_1C_) levels, LDL levels, and HDL levels and prevented the development of type 2 diabetes in high-risk adult subjects [16, 20, 21]. These results supported our findings in a pediatric population. The effect of lifestyle modification shown in this study could act as a pilot and serve as a reference for future high-quality randomized controlled trials.

The Institute of Medicine recommended “an ecological model” for public health interventions, in which the individual subject is viewed “within a larger context of family, community, and society” [46]. During the developmental stages, children require a high amount of energy to grow, making these stages more important than other stages. The American Academy of Pediatrics recently announced the “management of newly diagnosed type 2 diabetes in children and adolescents” [47]. Similar to the management of type 2 diabetes in adults [17], lifestyle modification and Metformin® were recommended as a first-choice therapy, except during hyperglycemic emergencies [47]. This aligns with our study findings that lifestyle modification interventions should be implemented before the onset of the disease. A recent randomized trial tested the dose-dependent benefits of exercise on insulin resistance and body fat in 222 overweight or obese children [48]. A meta-analysis pooled 49 studies on adults to form a conclusion on lifestyle modification programs, which included changing eating habits, consuming healthy food, and increasing fitness and physical activity; they found a reduction in BMI and FPG levels of adults [21]. This was similar to the findings of our study. We applied a subgroup analysis to overcome the high heterogeneity of BMI *z*-scores and insulin levels but not glucose levels. The intervention follow-up length was associated with the outcomes. Specifically, the metaregression revealed that lifestyle interventions were shown to improve FPG levels and BMI *z*-score but not insulin levels or HbA_1C_ changes. Perhaps the small sample size of such studies on children induced potential publication bias. Regardless of exercise intensity, we observed significant reductions in the risk of prediabetes compared with the control group.

The diagnostic criteria for diabetes mellitus in children are the same as those in adults [49]. FPG and HbA_1C_ have been both used as diagnostic criteria for diabetes mellitus in children [9]. However, the oral glucose tolerance test was difficult to perform in children. Other metabolic markers were also difficult to assess because not all of the studies evaluated these markers. This is one of the limitations of this study. This study was also limited by the lack of a standardized physical activity dosage, which made it difficult to compare each study and their reported effectiveness of lifestyle modification, possibly inducing higher heterogeneity. Unlike studies of adult populations, studies of pediatric populations additionally need to develop playful and age-appropriate intervention designs.

In adults with IGT or IFG, lifestyle modification programs have been consistently shown to be effective in the prevention of type 2 diabetes [17–20]. Our study demonstrated that lifestyle modification significantly reduced FPG levels in overweight/obese children. It should be considered that vacations might affect lifestyle regulation as the follow-up duration was often limited by the semester and school year changes. FPG levels could also be affected by seasonal changes or holidays [50, 51]. Additionally, during puberty, sexual hormones play a more significant role in changing FPG levels [9, 52, 53]. The results may be counterintuitive as the reduction in FPG levels, BMI *z*-score, and insulin levels did not provide a clinically significant meaning. Lifestyle modification interventions are still useful for teenagers. Encouraging exercise in this population is suggested; however, diet cannot be too strict for growing adolescents.

It must be noted that 8 of the studies in this review were conducted in high-income countries and that the sample population primarily consisted of Caucasian subjects. Only one study consisted of Chinese population, and this may have acted as a confounding factor as the prevalence of diabetes is dependent on ethnicity. It has to be noted that Asians are more vulnerable to diabetes, despite a lower body mass index on average [54–56]. Therefore, there is a need to design ethnically tailored strategies to modify the lifestyles of different populations. Because the factors affecting Asian diabetes patient are not always the same as those in high-income Western countries [54], we encourage the medical professionals in Asian countries to perform more studies that control for variability in ethnicity to reach a better conclusion.

Furthermore, a healthy lifestyle behavior and attitude will be effective for improving the glucose balance, so the promotion of healthy school policies is needed to be wildly implemented in school environment, such as providing healthier meals and achieving physical activity goals in different age students to prevent children to become obese adults [57]. In particular, type 2 diabetes is associated with obesity and plasma glucose level. Treatment should not only normalize hyperglycemia but also focus on lifestyle modification [58].

For the early prevention of type 2 diabetes among children, interdisciplinary teamwork may improve the intervention integrity. For example, school nurses could provide a regular screening and refer high-risk students to general practitioners and dialectologists. Multiple approaches should be used to maintain energy balance in developing children, including nutritional education and physical activity. We strongly recommend the introduction of playful gym classes or interactive programs into daily life to increase energy expenditure. Furthermore, future studies are needed to examine the dose-response intervention, the effect of follow-up duration, and the cost-effectiveness of lifestyle modification programs to prevent diabetes in overweight/obese children.

Some limitations merit attention. First, the included studies had heterogeneity of the interventions of life style modifications. Second, the studies reported in the meta-analysis were conducted in the age range including puberty. Hormonal changes and gender effect at puberty might affect insulin sensitivity and glucose metabolism. Third, majority of the included studies were conducted in Western countries. However, the effect of lifestyle modification shown in this study still could act as a pilot and serve as a reference for future high-quality randomized controlled trials.

## 5. Conclusion

Lifestyle modification can modestly reduce FPG in overweight/obese children. Further large-scale studies with longer follow-up duration are warranted to examine the effects of lifestyle modification on type 2 diabetes mellitus prevention, especially in Asian countries.

## Supplementary Material

Figure S1: The quality assessment of included studies.
Figure S2: Meta-analysis funnel plot.
Figure S3: Meta-regression scatter plot of variables by study period.

## Figures and Tables

**Table 1:** Characteristics of studies included in this meta-analysis.

1st author/yr	Location	Study design	Subjects					Intervention style	Study period
			BMI	IG *n*	CG *n*	Age yrs	Race		
Boudreau, 2013 [35]	Massachusetts, USA	RCT	>85th	23	18	9~12	Latino	Power Up class and culture-sensitive health coaching for children and their caregivers	6 months

Carrel, 2005 [36]	Wisconsin, USA	RCT	>95th	27	26	12 ± 0.5	NA	School-based, fitness-oriented gymnasium classes and nutritional education	9 months

Foster, 2010 [37]	USA	RCT	>85th	1160	1132	11~14	H: 58.6	The HEALTHY Study, a multicomponent, school-based program: nutrition, physical activity, behavioral knowledge and skills, communications and social marketing	3 years
							B: 17.3		
							W: 17.0		
							O: 7.1		

Johnston, 2007 [38]	Texas, USA	RCT	>85th	40	20	10~14	Mexican-American	Intervention was 4 sessions on healthy eating habits and trainer-led physical activity, while the control was self-help	6 months

Savoye, 2014 [39]	Connecticut, USA	RCT	>95th	38	37	11~14	NH: 31.5%	BB program-lifestyle modification twice/wk, 50 min exercise and 40 min nutrition/behavior modification class.	6 months
							W: 39.5		
							B: 29%		

Uysal, 2014 [42]	Germany	CT	>95th	484	533	9~13	West Germany	Obeldicks lifestyle intervention program that included physical activity, nutrition education, and behavior therapy to children and their family	12 months

Van Der Baan-Slootweg, 2014 [40]	Netherlands	RCT	>95th	41	39	13.9 ± 2.5	Dutch	Inpatient treatment compared with ambulatory lifestyle interventions. Hospitalized for 26 wks, exercised 45 min for 4 days/wk, nutrition and behavior program 60 min/wk	30 months

Vos, 2011 [41]	Netherlands	RCT	>95th	40	39	8~17	NE: 31.6%	Family-based multidisciplinary cognitive behavioral lifestyle intervention	12 months
							O: 68.4%		

Wang, 2015 [43]	Beijing, China	NRC	>85th	90	136	7~12	Chinese	The comprehensive intervention group for 6 cycle (2 months/cycle); the PA only group (Happy 10 program); the diet only group (nutrition education program for 80 mins); and a control group.	12 months

RCT: randomized controlled trial; CT: clinical trial design; NRC: nonrandomized cluster design; IG: intervention group; CG: control group, *Race*. H: Hispanic; B: Black; W: White; O: others; NE: Northern European; NA: not classified.

**Table 2:** Summary of the FPG level (mg/dL), BMI or BMI *z*-score, and insulin (mg/dL) level changes before and after the treatment between the intervention and control groups.

1st Author/yr	Fasting Plasma Glucose				BMI or BMI *z*-score				Insulin level			
	Intervention		Control		Intervention		Control		Intervention		Control	
	Before	After	Before	After	Before	After	Before	After	Before	After	Before	After
Boudreau, 2013 [35]	83.7 ± 17.3	84.2 ± 5.6	83.3 ± 4.1	85.5 ± 5.2	2.0 ± 0.3	2.0 ± 0.4	2.2 ± 0.4	2.1 ± 0.5	21.8 ± 17.3	24.2 ± 17.8	11.5 ± 4.9	13.2 ± 4.6
Carrel, 2005 [36]	83 ± 4	87 ± 5	86 ± 7	93 ± 7	32 ± 6	33 ± 10	30 ± 4	30 ± 5	25.7 ± 15.7	32.6 ± 6.4	31.8 ± 5	32.5 ± 6
Foster, 2010 [37]	94.1 ± 6.5	94.0 ± 7.9	94.0 ± 6.9	94.9 ± 8.2	1.80 ± 0.44	1.62 ± 0.61	1.80 ± 0.44	1.66 ± 0.59	17.9 ± 12.5	21.5 ± 18.2	18.5 ± 15.0	22.6 ± 16.1
Johnston, 2007 [38]	−0.24 ± 0.46		−0.41 ± 0.45		−0.99 ± 3.79		1.08 ± 1.00		−6.22 ± 11.95		−5.00 ± 17.51	
Savoye, 2014 [39]	−0.5 (−3.1, 2.0)		2.5 (0.7, 5.7)		−0.05 (−0.09, −0.01)		0.04 (0.00, 0.08)		−4.9 (−10.0, 1.1)		5.2 (−1.3, 12.9)	
Uysal, 2014 [42]	86 (81, 90)	86 (82, 91)	87 (83, 91)	87 (83, 93)	2.42 (2.12, 2.72)	2.25 (1.89, 2.61)	2.45 (2.19, 2.80)	2.48 (2.13, 2.83)	14 (9, 22)	13 (8, 19)	15 (10, 23)	15 (10, 22)
Van Der Baan-Slootweg, 2014 [40]	78.4 ± 9.9	74.4 ± 11.6	76.6 ± 6.3	77.1 ± 11.5	3.35 ± 0.4	2.84 ± 0.7	3.35 ± 0.4	3.05 ± 0.8	16.1 ± 6.6	10.2 ± 16.0	18.3 ± 8.7	16.6 ± 15.8
Vos, 2011 [41]	95.4 ± 7.25	91.8 ± 3.6	93.6 ± 7.20	91.8 ± 5.4	4.2 ± 0.7	3.8 ± 1.2	4.3 ± 0.6	4.2 ± 0.7	19.5 ± 12.2	20.5 ± 10.7	14.9 ± 8.0	18.8 ± 6.3
Wang, 2015 [43]	98 ± 12	101 ± 12	73 ± 14	91 ± 6	24.48 ± 3.03	25.28 ± 3.05	22.25 ± 2.78	23.29 ± 2.94	ND		ND	

Values are presented as the mean ± SD, or 95% confidence interval. NS: not significant; BMI: body mass index.

**Table 3:** Summary of the HbA_1C_  [%] changes before and after the treatment between the intervention and control groups.

1st author/yr	HbA_1C_ [%]			
	Intervention		Control	
	Before	After	Before	After
Boudreau, 2013 [35]	5.6 ± 0.3	5.7 ± 0.3	5.6 ± 0.3	5.7 ± 0.3
Savoye, 2014 [39]	−0.16 (−0.27, −0.05)		−0.05 (−0.17, 0.07)	
Uysal, 2014 [42]	5.4 (5.2, 5.7)	5.5 (5.3, 5.7)	5.5 (5.3, 5.7)	5.5 (5.3, 5.8)
