# Supplementary material for: A Systematic Review and Meta-Analysis of the Effect of Lifestyle Modification on Metabolic Control in Overweight Children
Source: Evid Based Complement Alternat Med. 2017 Sep 27;2017:5681909. doi: 10.1155/2017/5681909 (PMC5635284; doi:10.1155/2017/5681909)
Supplement: Supplementary file 1 — Figure S1: The quality assessment of included studies. Figure S2: Meta-analysis funnel plot. Figure S3: Meta-regression scatter plot of variables by study period. [file 5681909.f1.docx]

**Figure S1. The quality assessment of included studies**

1. **
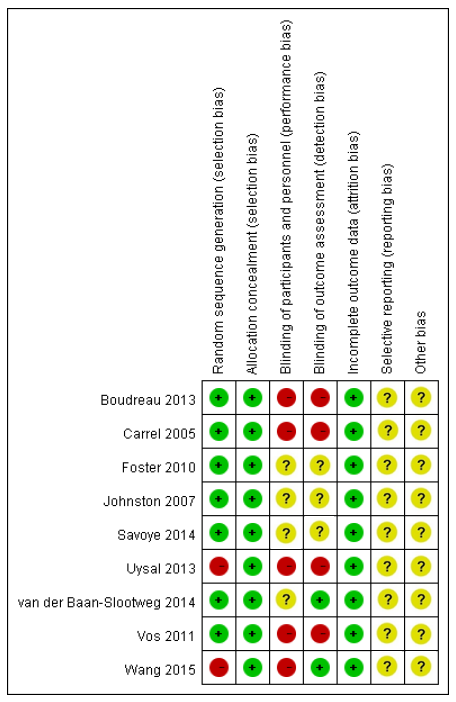
**Risk of bias summary

(B) Risk of bias graph

**
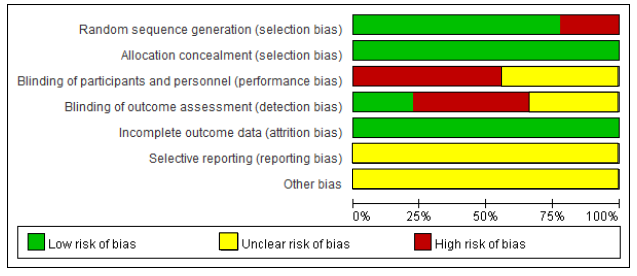
**

**Figure S2.** Meta-analysis funnel plot

1. Fasting plasma glucose


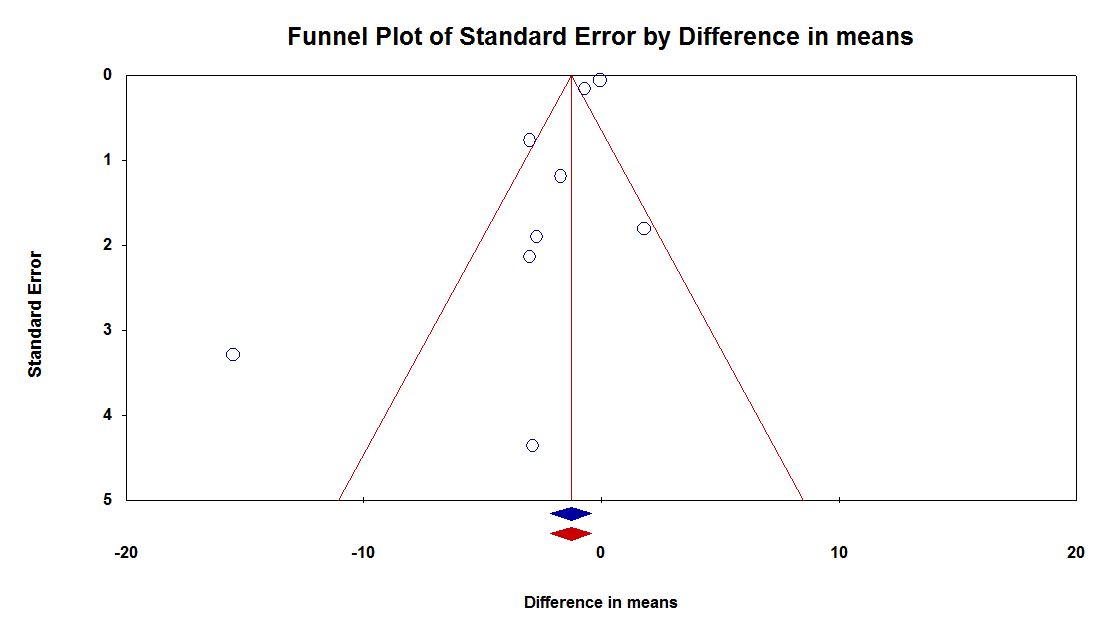


1. BMI z-score


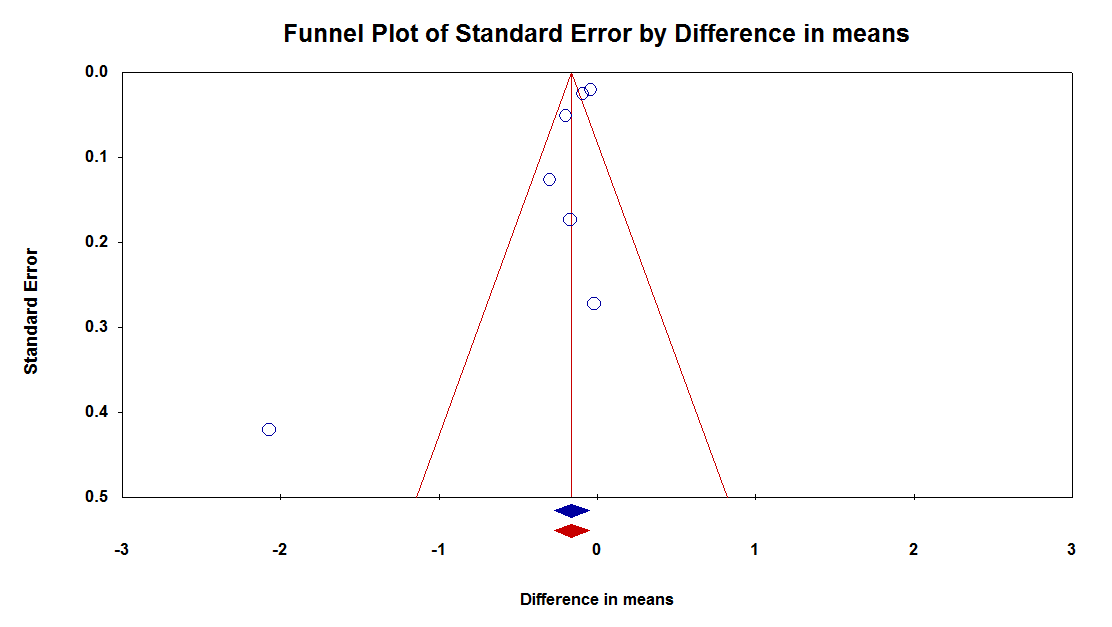


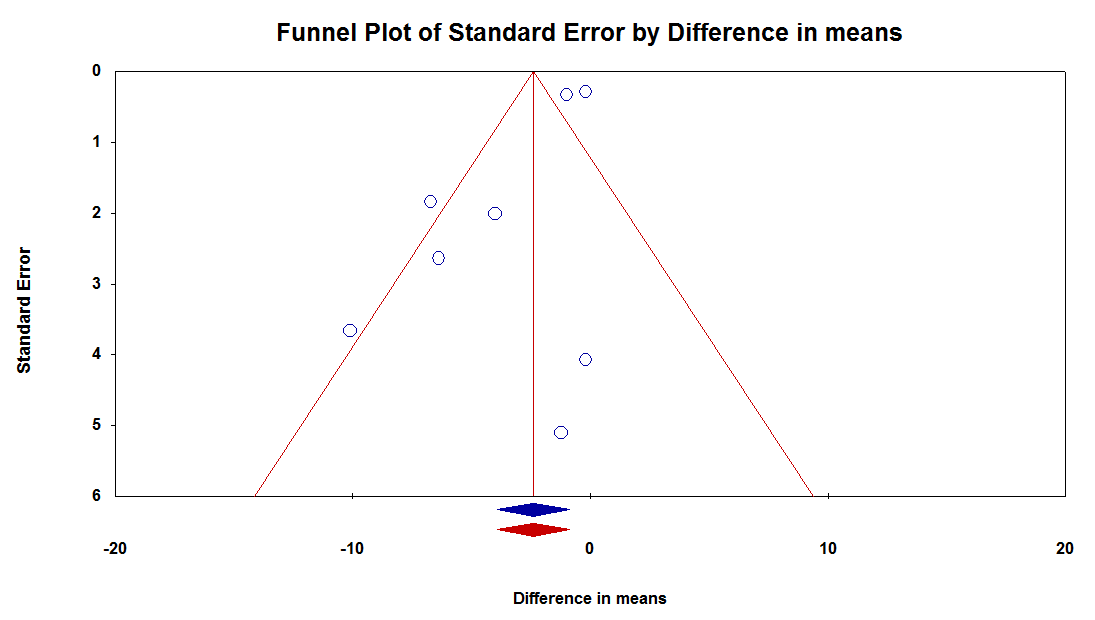
(C) Fasting insulin level

1. HbA1c


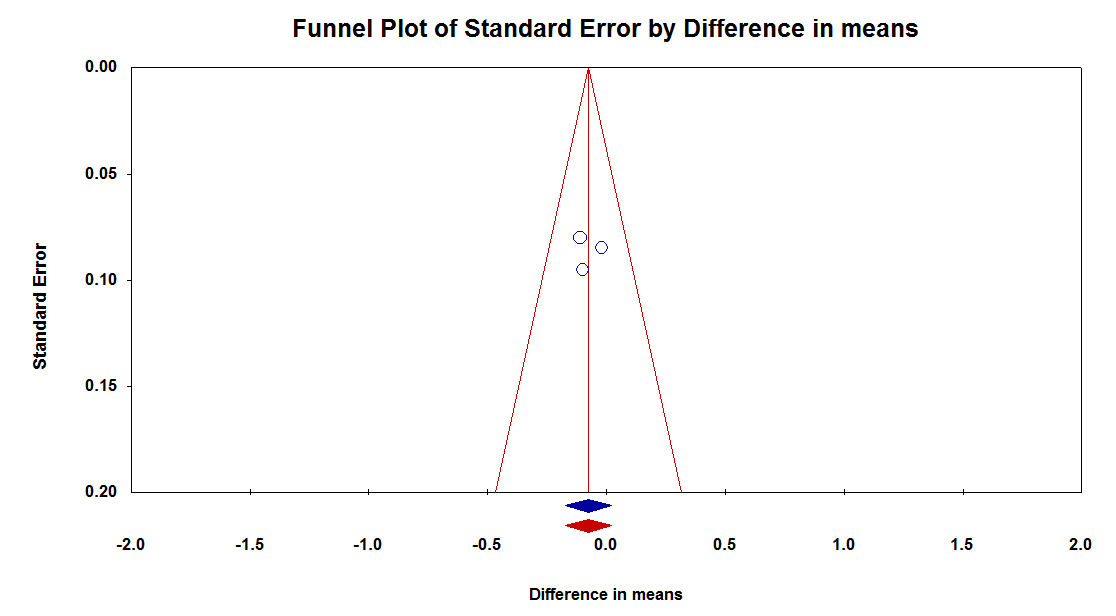


**Figure S3.** Meta-regression scatter plot of variables by study period

(A) Fasting plasma glucose

Tau^2^ =5.95, Q-value=59.25, p-value=0.000

(B) BMI z-score

Tau^2^ =0.03, Q-value=35.40, p-value=0.000

(C) Fasting insulin level

Tau^2^ =8.71, Q-value=28.52, p-value=0.000
